# Genome-wide association studies for soybean epicotyl length in two environments using 3VmrMLM

**DOI:** 10.3389/fpls.2022.1033120

**Published:** 2022-11-14

**Authors:** Huilong Hong, Mei Li, Yijie Chen, Haorang Wang, Jun Wang, Bingfu Guo, Huawei Gao, Honglei Ren, Ming Yuan, Yingpeng Han, Lijuan Qiu

**Affiliations:** ^1^ Key Laboratory of Soybean Biology in Chinese Ministry of Education (key Laboratory of Soybean Biology and Breeding/Genetics of Chinese Agriculture Ministry), Northeast Agricultural University, Harbin, China; ^2^ Institute of Crop Science, National Key Facility for Crop Gene Resources and Genetic Improvement (NFCRI) Chinese Academy of Agricultural Sciences, Beijing, China; ^3^ Crop Information Center, College of Plant Science and Technology, Huazhong Agricultural University, Wuhan, China; ^4^ College of Agriculture, Yangtze University, Jingzhou, China; ^5^ Jiangsu Xuhuai Regional Institute of Agricultural Sciences, Xuzhou, China; ^6^ Nanchang Branch of National Center of Oil crops Improvement, Jiangxi Province Key Laboratory of Oil crops Biology, Crops Research Institute of Jiangxi Academy of Agricultural Sciences, Nanchang, China; ^7^ Soybean Research Institute, Heilongjiang Academy of Agricultural Sciences, Harbin, China; ^8^ Qiqihar Branch of Heilongjiang Academy of Agricultural Sciences, Qiqihar, China

**Keywords:** genome-wide association analysis, single nucleotide polymorphism, candidate genes, 3VmrMLM, epicotyl length

## Abstract

Germination of soybean seed is the imminent vital process after sowing. The status of plumular axis and radicle determine whether soybean seed can emerge normally. Epicotyl, an organ between cotyledons and first functional leaves, is essential for soybean seed germination, seedling growth and early morphogenesis. Epicotyl length (EL) is a quantitative trait controlled by multiple genes/QTLs. Here, the present study analyzes the phenotypic diversity and genetic basis of EL using 951 soybean improved cultivars and landraces from Asia, America, Europe and Africa. 3VmrMLM was used to analyze the associations between EL in 2016 and 2020 and 1,639,846 SNPs for the identification of QTNs and QTN-by-environment interactions (QEIs)”.A total of 180 QTNs and QEIs associated with EL were detected. Among them, 74 QTNs (ELS_Q) and 16 QEIs (ELS_QE) were identified to be associated with ELS (epicotyl length of single plant emergence), and 60 QTNs (ELT_Q) and 30 QEIs (ELT_QE) were identified to be associated with ELT (epicotyl length of three seedlings). Based on transcript abundance analysis, GO (Gene Ontology) enrichment and haplotype analysis, ten candidate genes were predicted within nine genic SNPs located in introns, upstream or downstream, which were supposed to be directly or indirectly involved in the process of seed germination and seedling development., Of 10 candidate genes, two of them (Glyma.04G122400 and Glyma.18G183600) could possibly affect epicotyl length elongation. These results indicate the genetic basis of EL and provides a valuable basis for specific functional studies of epicotyl traits.

## Introduction

Epicotyl length (EL), an important complicated and agronomically trait, was significantly related to plant density and sowing depth of soybean (Camargos et al., 2019). EL exhibited the higher genetic variability at the early developmental stages of soybean, especially at V_2_ and V_3_ development stages (Matsuo et al., 2012). EL also affected plant height and yield of soybean (Hanyu et al., 2020). As a typical quantitative trait, EL, with relatively high heritability (more than 95%), was controlled by a few large-effect genes and a series of polygenes (Chaves et al., 2017). EL was significantly affected by environment, genotype their interactions (Chaves et al., 2017; Hanyu et al., 2020). Several studies showed that genetic and environmental variation approximately accounted for half of experimental observation. Although EL has been considered as the important feature of variety during the long-term soybean breeding, development of soybean cultivar with reasonable and stable EL through traditional selection method was still difficult (Chaves et al., 2017). It required evaluation in multiple environments over several years, and traditional selection method was expensive, time-consuming and labor-intensive (Chaves et al., 2017).

Molecular marker could effectively improve traditional selection efficiency by increasing the allele’s frequency of desirable quantitative trait loci (QTLs). Presently, linkage analysis and association analysis, were two major strategies utilized to identify QTLs of important traits in crops (Li et al., 2020; Liu et al., 2020; Wang et al., 2021). Segregating population based linkage analysis strategy is a well-known approach to obtain QTLs, followed by fine mapping using larger secondary population or other types of population with sufficient map resolution, then candidate genes could be cloned for functional characterization. (Dinka et al., 2007) mapped four additive QTLs for the length of hypocotyl in soybean. However, none of EL QTLs of soybean has been reported to date. Based on diversegermplasms, Genome-Wide Association Study (GWAS) take advantages of historical recombination events offered another strategy to effectively fine map QTL with rapid decay of linkage disequilibrium (LD) (Flint-Garcia et al., 2003). Due to the advances in next-generation sequencing (NGS) technologies or Chip with high-density SNPs, GWAS has been widely extensively utilized to dissect genetic architecture of important traits in crops including soybean, e.g. biotic stress (Zhao et al., 2015; Zhao et al., 2017), abiotic stress (Zhang et al., 2015; Jia et al., 2017), yield-related trait including seed weight (Yan et al., 2017), maturity time (Contreras-Soto et al., 2017), and seed composition including seed oil content (Cao et al., 2017; Li et al., 2018), seed protein content (Zhang et al., 2019), tocopherol (Sui et al., 2020) and isoflavone concentration (Wu et al., 2020). Liang et al. (2014) identified four additive QTLs for the length of hypocotyl in soybean using linkage analysis. However, no EL QTLs in soybean has been reported to date.

Since the establishment of mixed linear model (MLM) method in genome-wide association studies (GWAS) (Zhang et al., 2005; Yu et al., 2006; Kang et al., 2008), these methods have proven to be useful in controlling for population structure and relatedness of individuals. However, these methods are computationally challenging for large datasets. Thus, a series of fast MLM-based algorithms have been developed and widely-used, such as CMLM (Zhang et al., 2010), EMMAX (Kang et al., 2010), FaST-LMM (Lippert et al., 2011), and GEMMA (Zhou and Stephens, 2012). In these methods, single marker genome scanning was used to identify significant QTNs. This is involved in multiple tests. To control false positive rate, Bonferroni correction is frequently adopted. The stringent significant criterion frequently results in the missing of some important loci, especially in crop GWAS. To overcome this issue, several multi-locus mixed model methods have been proposed and widely used (Segura et al., 2012; Wang et al., 2016; Wen et al., 2017). As we know, there are frequently three genotypes for each marker in GWAS. Two effects should be estimated, while their polygene backgrounds should be controlled. In most GWAS methods, however, only one confound effect is estimated, while its polygene background is controlled. To solve this issue, recently, Li et al. (2022b) established a three-variance-component mixed linear model framework, 3VmrMLM, to identify QTNs, QTN-by-environment interactions (QEIs), and QTN-by-QTN interactions under controlling all the possibly polygene backgrounds.

Cytokinins and light can sometimes elicit similar morphological and biochemical responses. In the absence of light plant seedlings have long epi- or hypocotyls and appressed leaves with the plastid development blocked at the stage of etioplasts or amyloplasts. The l6 ight-i6 ndependent p6 hotomorphogenesis (lip1) mutant of pea shows many of the characteristics normally associated with light-grown seedlings when grown in complete darkness, such as expanded leaves, a short epicotyl and partially developed chloroplast (Frances et al., 1992). Chory et al. the effects of cytokinin treatment on epicotyl growth inhibition of lip1 i n darkness are comparable to a hypocotyl growth inhibition observed in Arabidopsis(Chory et al., 1994), It appears that the effect of cytokinin on the growth of the axis of young hypogeal (e.g., Arabidopsis) and epigeal (e.g., pea) seedlings is similar. The phenotype of wild-type Arabidopsis plants following cytokinin treatment is similar to that of the amp1 mutant of Arabidopsis, suggesting that light and cytokinin act through a common signaling pathway (Chory et al., 1994; Seyedi et al., 2001). genetic analysis of Arabidopsis has provided unequivocal evidence that the brassinosteroids(BRs) are essential phytohormones (He et al., 2003). Brassinolide (BL), an end product of campesterol oxidationis is required for the regulation of cell elongation, stress response, male fertility, pigment biosynthesis, and numerous other developmental and physiological responses in higher plant (Grove et al., 1979), The Arabidopsis CYP90A1 (constitutive photomorphogenesis and dwarfism, CPD) has been identified to functions as the C-23 hydroxylase in the biosynthetic pathway of brassinosteroids, and cpd mutant exhibited the most pronounced effect in dwarf phenotype than another five cytochrome P450 mutants. The biosynthetic model of BRs has been clearly identified in *Arabidopsis*, we supposed a similar model, It has been proved in 1998 that the transcription of *Arabidopsis* CYP90A1 was negatively controlled by exogenous brassinolide (Mathur et al., 1998).

To address above mentioned issues, 951 landraces and cultivarsselected from Chinese primary core collection in the Chinese National Soybean GeneBank (CNSGB), were phenotyped for EL in 2016 and 2020, and genotyped by 1,639,846 SNPs in order to identify QTNs, QEIs, and their candidate genes for EL in soybean.

## Materials and method

### Plant materials, filed trials and epicotyl length evaluations

To construct a diversity panel of EL, a total of 951 landraces was selected from more than 20,000 samples, which delegated much of the representatives of diversity of the collection at the Chinese National Soybean GeneBank (CNSGB). These tested materials were planted with the single row plots (3-m long and 0.35-m between rows), which was performed with the completely randomized design and three replications in Sanya, Hainan China in 2016 and 2020.

A total of 3 randomly selected plants from each plot were phenotyped for EL by measuring the distance between the cotilenodary knot and the unifoliate leaves pair knot using vernier caliper.

### DNA isolation and genome sequencing

The genomic DNA of each tested samples were isolated from fresh leaves of a single plant, and then resequenced. Sequencing libraries were constructed based on TruseqNano^®^ DNA HT sample preparation Kit (Illumina USA), and index codes were added to attribute sequences to each accession according to the method described by (Li et al., 2020a). The Illumina Hiseq X platform was used to analyze the libraries of these samples. A total of 10.58 Tb raw sequences with 150-bp read length, were obtained. After sequence quality filtering, the clean read of all tested samples, were aligned to soybean reference genome *via* Short Oligonucleotide Alignment Program 2 (SOAP2) software. The SNPs were calling based on MAF ≥ 0.05. The genotype was regarded as heterozygous if the depth of minor allele/the total depth of the sample was more than 1/3.

### Population structure evaluation and linkage disequilibrium (LD) analysis

The population structure of GWAS panel were evaluated based on principle component analysis (PCA) programs of Software package GAPIT (Lipka et al., 2012). LD was called with SNP (MAF ≥ 0.04 and missing data ≦ 10%) based on TASSEL version 3.0 (Bradbury et al., 2007).

### Association analysis of epicotyl length of soybean

A total of 1,639,846 SNPs from 951 landraces samples were utilized to detect association signals of EL in soybean. Imputed genotype of total sample panel was first transformed in to *.fam, *.bed, and *.bim format, ELS and ELT in two different environments were adopted as phenotype, evolutionary population structure encoded as B (Landrace) and C (Improved cultivar), and kinship were employed as covariates for multi-environment joint analysis with significant level of 0.01 using IIIVmrMLM software of Li et al. (2022b); Li et al. (2022c). Linkage disequilibrium (LD) of 250kb up- and down-stream of significantly associated SNP were calculated by PLINK1.9, and threshold of regional average LD > 0.9 was used to define credible associated region. Functional annotation of candidate genes was performed based on annotation by phytozome (https://phytozome-next.jgi.doe.gov/info/Gmax_Wm82_a2_v1).

### *Definition and* verification of candidate genes

Then SNP variations in the coding region of candidate genes were analyzed to screen candidate genes with mutation type of nonsynonymous, stoploss, stopgain, or alternative splicing. To further screen candidate genes, fixation index (F_ST_) was calculated by published genome sequences data of 2214 soybeans (Li et al., 2022d) using vcftools (0.1.13) with window size of 100bp, and coding regions with F_ST_ ≥ 0.6 were regarded as potential domestication gene (Song et al., 2013). Subsequently, spatial and temporal expression of candidates were analyzed using publicly available soybean transcriptome integration dataset (Yu et al., 2022). Functional annotations of all candidate genes were performed based on the SoyBase database (http://www.soybase.org) and the Kyoto Encyclopedia of gene and genomes (KEGG).

### Haplotype analysis

Gene region were defined using *.gff, regional genotype of hapmap diploid were extracted from imputed genotype, then haplotypes were inferred based on regional genotype classified according to its location relative to the gene structure. Significance of traits between different haplotypes were performed by Kruskal-Wallis (*P*<0.01) (Theodorsson, 1986). Haplotype TCS network was inferred using PopART (Bandelt et al., 1999; Clement et al., 2002; French et al., 2014). Geographic mapping of different haplotypes was performed using R scripts.

## Results

### Distribution of the landraces used in the experiment

**Figure 1:**
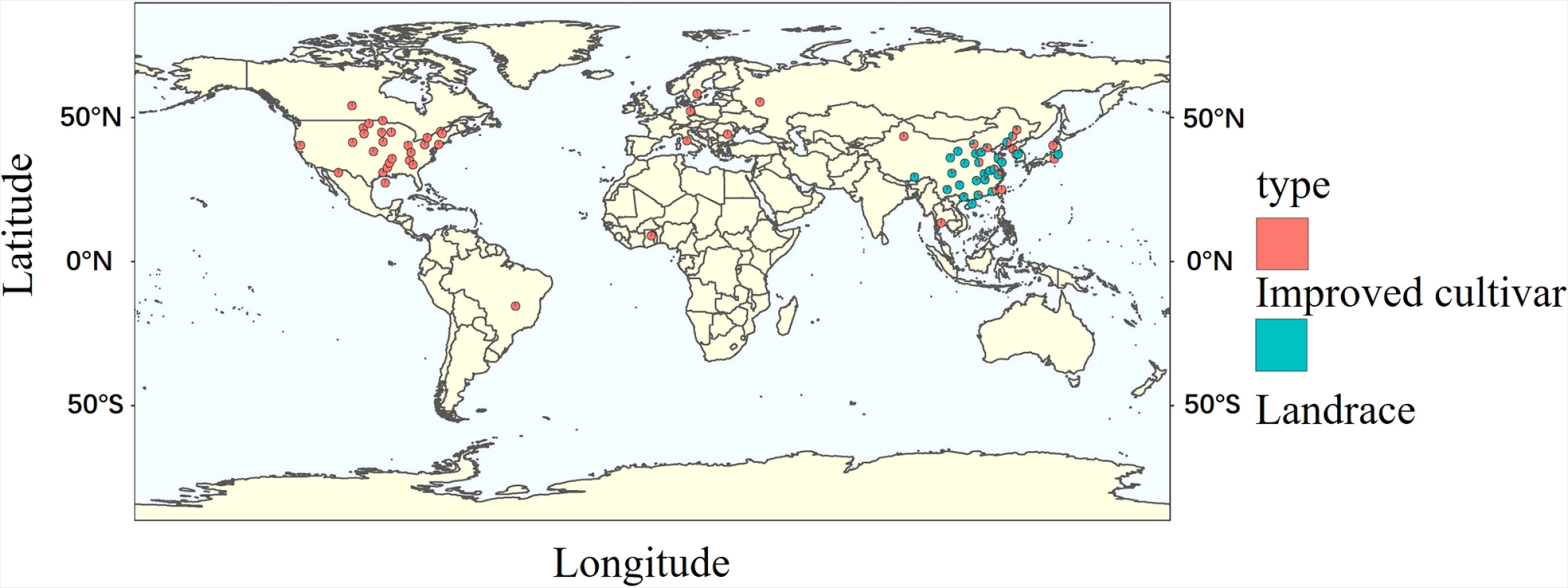
The geographical distribution of the tested accessions.

**Figure 2:**
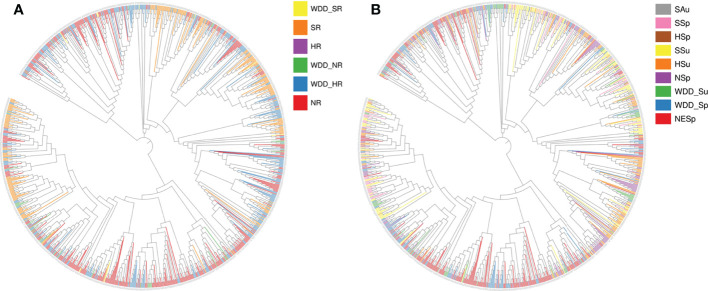
951 species construct phylogenetic tree according to geographical distribution and ecological type. **(A)** Variety Geographical Distribution Evolutionary Tree. WDD_: Oversea_; NR: Northern Region; HR: Central Region; SR: Southern Region **(B)** Variety Ecotype Evolutionary Tree. SAu, Southern autumn soybean; SSp, Southern spring soybean; HSp, Huanghuai summer soybean; SSu, Southern summer soybean; HSu, Huanghuai summer soybean; NSp, Northern spring soybean; NESp, Northeast Spring Soybeans; WDD_Su, Oversea summer soybean; WDD_Sp, Oversea spring soybean.

Globally, the improved cultivars selected for the experiment mainly comes from America and Asia, with few from Europe and Africa. Landraces were all obtained from Asia (**Figure 1**). To better understand the genetic architecture of these germplasms, geographical distribution and ecological types were taken into account for classification. Both domestic and foreign varieties can be divided into southern (SR), northern (NR) and central (HR) varieties, namely domestic varieties (SR, HR, NR) and foreign varieties (WDD_SR, WDD_HR, WDD_NR). Domestic NR sources are the maximum, and foreign WDD_HR varieties account for more than half of the total foreign varieties (**Figure 2A** and **Table S1**). According to ecological types, domestic cultivars can be divided into northeast spring type (NESp), northern spring type (NSp), Huang-huai spring type (HSp), Huang-huai summer type (HSu), Southern spring type (SSp), Southern summer type (SSu) and Southern autumn type (SAu), with NESp ranking the first place. The selected foreign varieties were mainly divided into spring type (WDD_Sp) and summer type (WDD_Su), and the quantity of WDD_Sp was twice as much as WDD_Su (**Figure 2B** and **Table S2**). These results demonstrated that nearly 80% of the varieties used in the experiment came from China, and 60% of the varieties obtained abroad were spring varieties in the central region.

### Statistical analysis for inflorescence length of the association panel

The EL of 951 landraces in Sanya, Hainan China in 2016 and 2020, were evaluated, respectively. The skewness and kurtosis of EL the three environments were less than ±1, which exhibited a continuous variation and the near normal distribution (**Table S3**). Therefore, EL of the association panel in this study, were appropriate.

### Distribution of SNPs and analysis of mapping population

**Figure 3:**
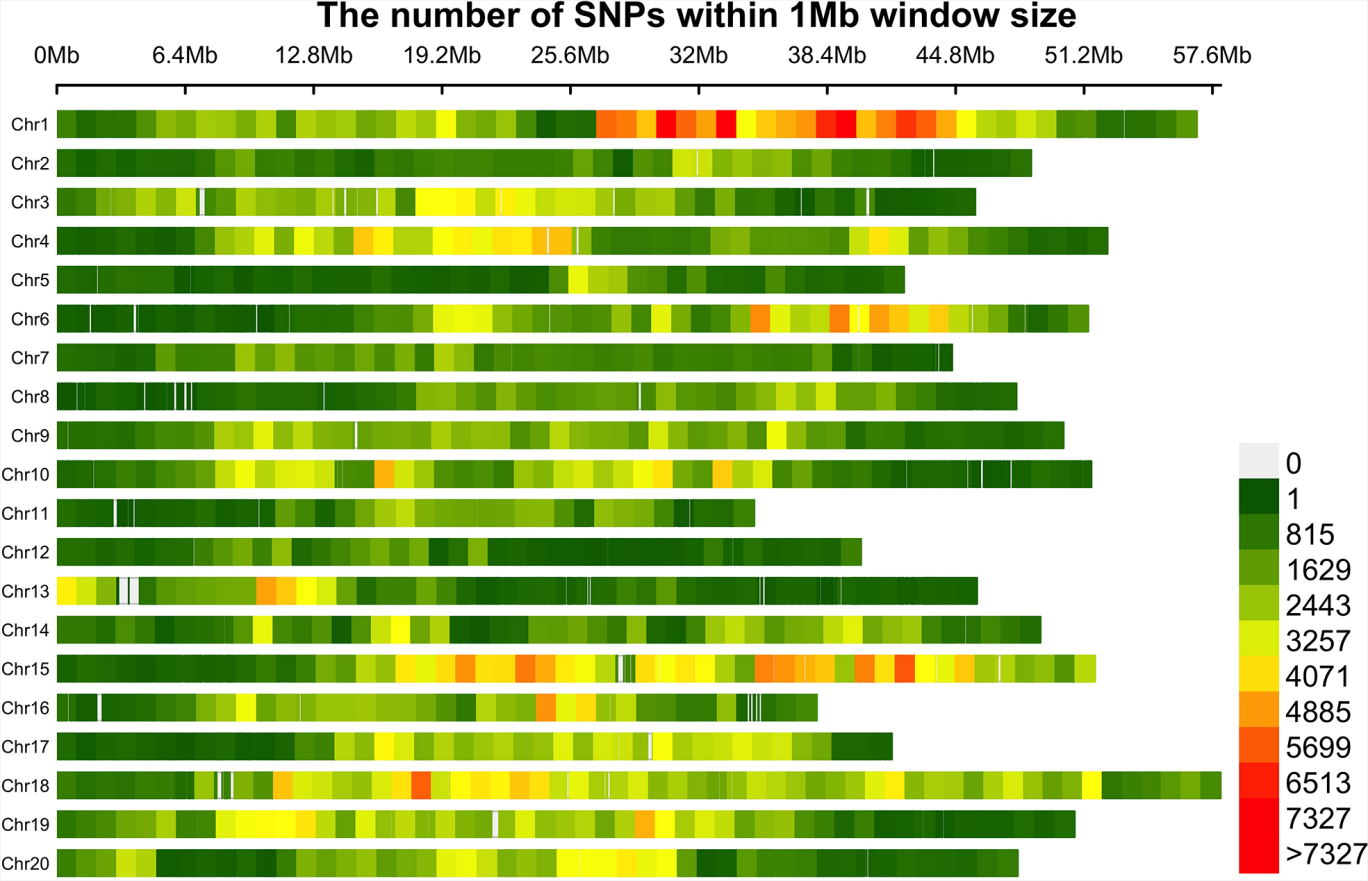
Distribution of SNP markers among 20 chromosomes.

**Figure 4:**
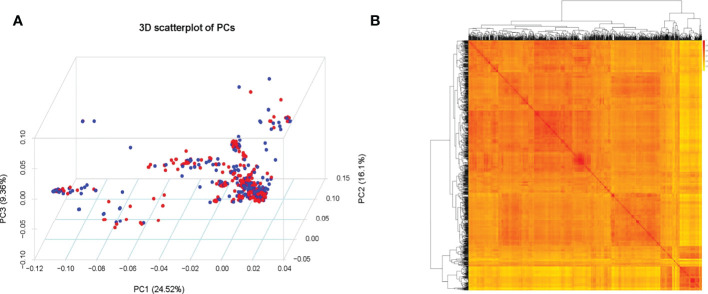
**(A)** Population structure of soybean germplasm. **(B)** Heatmap of the kinship matrix of the 951 soybean accessions.

**Figure 5:**
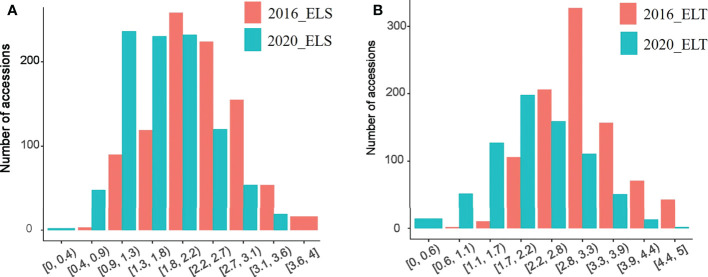
ELS and ELT phenotype distriution. **(A)** ELS phenotypes at different ages **(B)** ELT phenotypes at different ages.

Based with the frequency > 0.05 as the minor allele and the missing data less than 0.03, a total of 1,639,846 single nucleotide polymorphisms (SNPs) were unevenly distributedon 20 chromosomes of soybean genome. with a density of578.8 bp per SNP on average, and varied from 337.3bp~1334.4bp per SNP. In detail, there were 168,498 SNPs on Chr1 with the highest density (337.3bp/SNP), 31,650 SNPs on Chr5 with lowest density (1334.4bp/SNP). (**Figure 3**). Based on these SNPs, principal component analysis and phylogenetic analysis were performed on the association panel. The results showed that the first PCs explained 24.52% of the genetic variation, the 951 varieties were divided into two categories with apparent discrepancy of genetic relatedness (**Figure 4**). For a preferably clearer study of epicotyl traits, they were also divided into two categories, ELS and ELT. Statistical methods were used to test that ELS and ELT showed normal distribution in different environments among varieties (**Figure 5**).

### Quantitative trait nucleotide associated with epicotyl length-related traits by GWAS

**Figure 6:**
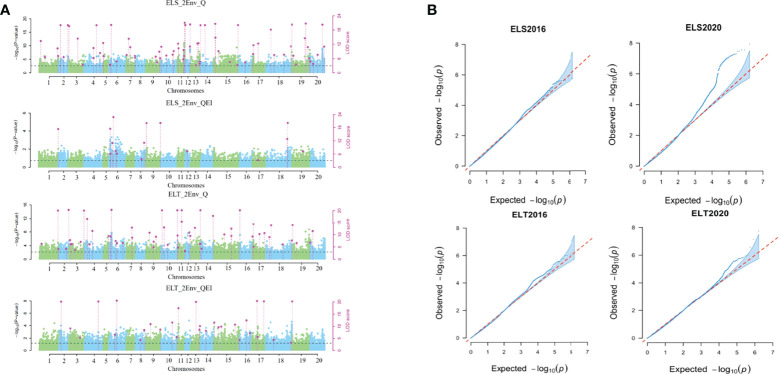
Results of association mapping of soybean epicotyl length traits. **(A)** Manhattan plot of locus distribution; **(B)** phenotype fitting results.

QTN (Q) and QTN-by-environment interaction (QEI) detection method in the 3VmrMLM was used to analyze SNP-trait associations in two EL two-environment datasets, ELS (2016 and 2020) and ELT (2016 and 2020). A total of 180 QTNs and QEIs associated with epicotyl length were detected. Among them, 74 QTNs (ELS_Q) and 16 QEIs (ELS_QE) were identified to be associated with ELS, and 60 QTNs (ELT_Q) and 30 QEIs (ELT_QE) were identified to be associated with ELT. **Figure 6** Of these, three sites (Gm_09_28400545, Gm_11_31100989, Gm_19_557643) could be found in all these four result datasets (**Table S4**).

### Prediction of candidate genes for epicotyl length traits

**Figure 7:**
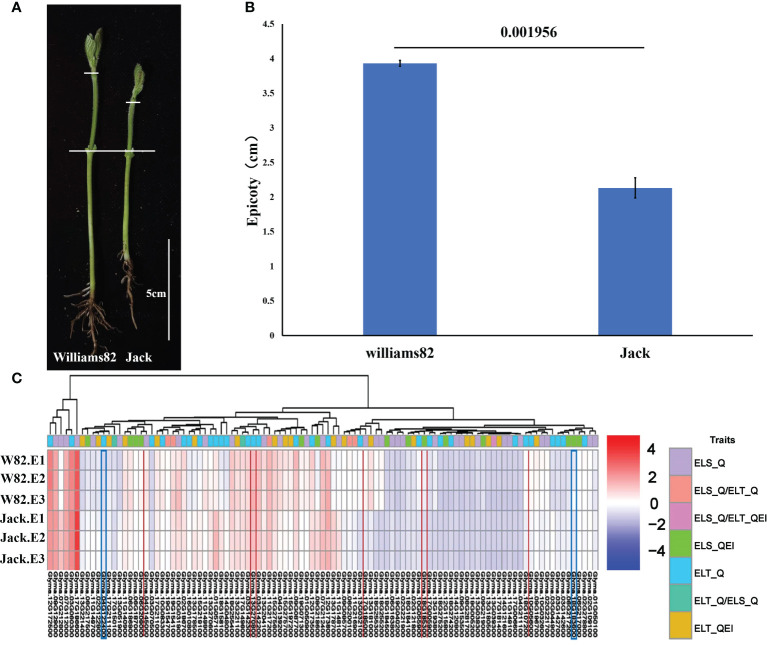
Epicotyl length of Williams82 and Jack and expression analysis of 103 candidate genes. **(A)** Epicotyl phenotype of W82 and Jack **(B)** Epicotyl Length Analysis of W82 and Jack **(C)** Transcriptome alignment of 103 candidate genes.

We performed candidate gene prediction analyses with peak SNP of ±100 kb based on the physical locations of 180 SNPs associated with epicotyl length. A total of 1945 genes were included in these regions (**Table S4**). Functional annotation of 1945 genes were completed by using *Arabidopsis* annotation information. site contribution rate, Transcription abundance of candidate genes in epicotyl of two representative soybean germplasms including cultivar Williams 82 with a long epicotyl of 3.93 cm and cultivar Jack with a short epicotyl of 2.13 cm were analyzed using publicly available soybean transcriptome integration dataset (Yu et al., 2022). By comparing the epicotyl lengths of Williams 82 and Jack, a very significant difference was found (**Figures 7A, B**). Based on the transcriptome data of epicotyls from Williams 82 and Jack, 585 out of 1945 genes were not expressed in both epicotyls of Williams 82 and Jack, 94 genes were expressed only in the epicotyl of Jack and 60 genes were expressed only in the epicotyl of Williams 82. A total of 1206 genes were expressed in both epicotyls of Williams 82 and Jack, of them, 157 genes were significantly differentially expressed in Williams 82 and Jack. Combined with Arabidopsis annotation information, 103 genes were identified as potentially candidate genes for epicotyl length (**Table S5**, **Figure 7C**). These differentially expressed genes in long and short epicotyl cultivars might be related to the length of epicotyl of soybean.

**Figure 8:**
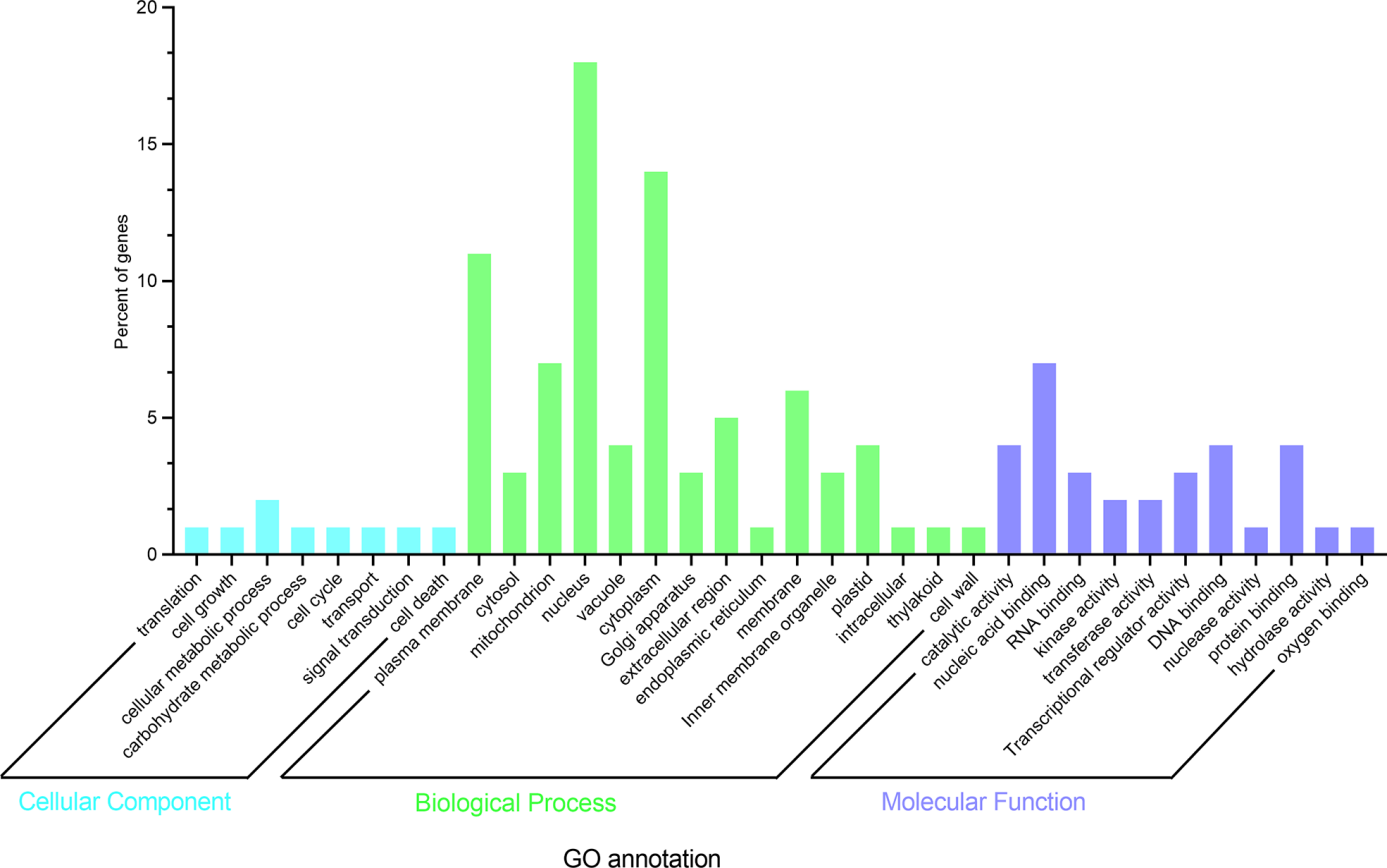
Functional categories of the genes in 100kb flanking regions around peak SNPs.

To further elucidate whether the differentially expressed genes were related to the length of the epicotyl, GO enrichment analysis was performed (http://amigo.geneontology.org/). GO enrichment analysis showed all genes were assigned to one of three GO categories: biological process (BP), molecular function (MF), and Cellular component (CC) (**Figure 8**).

Further, haplotype analysis was performed for 103 potentially candidate genes screened by the above analysis. epicotyl

**Figure 9:**
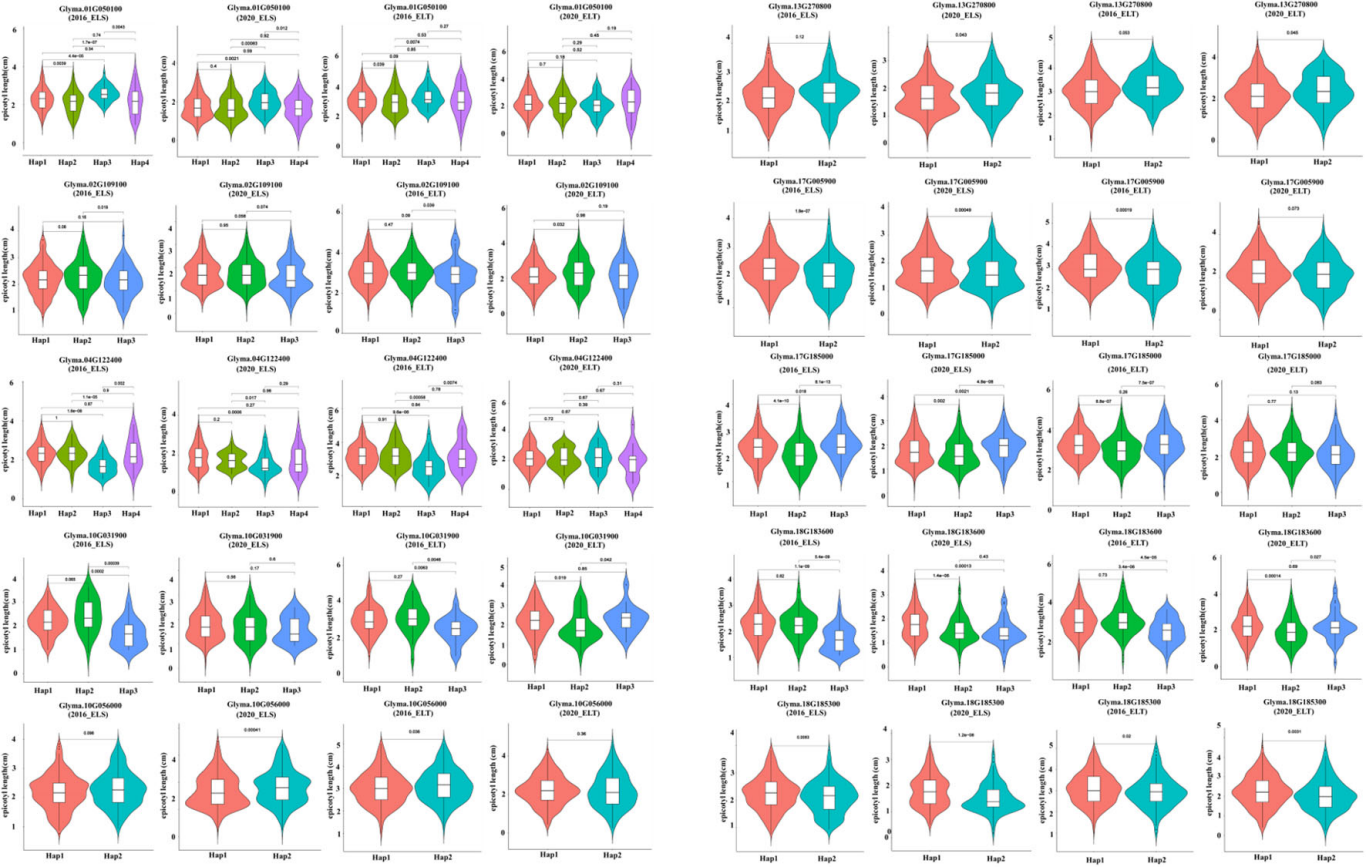
Genotyping of potential gene.

**Figure 10:**
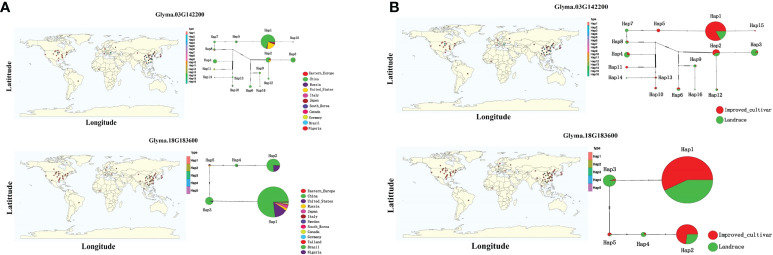
Haplotype analysis of candidate genes **(A)** distribution of geographical origin **(B)** distribution of cultivar characteristics.

In order to determine the role of the selected potential genes in soybean epicotyl growth, 22 potential candidates were screened by combining gene GO annotation and transcriptome differential expression analysis, and referring to Arabidopsis annotation information. Haplotype analysis identified 10 significantly different genesepicotyl. The Hap1 and Hap2 of Glyma.01G005900 in different years of ELS(*P*=0.0039) and ELT (*P*=0.039)showed extremely significant differences (P<0.01). The Hap1 and Hap3 of Glyma.18G183600(2016_ELS *P*=1.1e-09; 2020_ELS *P*=0.00013; 2016_ELT *P*=3.4e-06; 2020_ELT *P*=0.69), Glyma.18G185300(2016_ELS *P*=0.0083; 2020_ELS *P*=1.2e-08; 2016_ELT *P*=0.02; 2020_ELT *P*=0.0031), exhibited extremely significant differences (*P*<0.01), while the Hap1 and Hap3 of Glyma.01G050100(2016_ELS *P*=4.4e-05; 2020_ELS *P*=0.0021), Glyma.04G122400(2016_ELS *P*=1.6e-08; 2020_ELS *P*=0.0006), Glyma.18G183600(2016_ELS *P*=1.1e-09; 2020_ELS *P*=0.00013) in different years of ELS had a very significant difference in 2016 (*P*<0.01), but there was no significant difference in 2020. The candidate gene *Glyma.18G185300* showed a very significant difference in the two years of EL (*P*<0.01), and the ELT revealed a significant difference in 2016(2016_ELT *P*=0.02) and showed a very significant difference in 2020(2020_ELT *P*=0.0031) (**Figure 9**). Meanwhile, we counted the variation sites of 10 gene haplotypes (**Table S7**). The results demonstrated that *Glyma.04G122400*, *Glyma.10G031900* and *Glyma.18G183600* exist in exon variation sites, of which *Glyma.04G122400* and *Glyma.18G183600* exist non-synonymous mutations, hence, we speculate that *Glyma.04G122400* and *Glyma.18G183600* are candidate genes for epicotyl differences. At the same time, we combed the geographical origin of the two gene haplotypes and the distribution of variety characteristics. From the geographical distribution, we could see that Hap1, Hap2, Hap3 and Hap4 haplotypes of the two candidate genes were absolutely dominant in the selected varieties. In terms of ecological characteristics of cultivars, Hap1 and Hap2 haplotypes of the two genes accounted for more than Landrace haplotypes in improved cultivars (**Figure 10**).

We predicted ten plant growth-related genes, namely *Glyma.03G142200* (Ribosomal protein S10p/S20e family protein), *Glyma.04G122400* (DCD domain protein), *Glyma.04G145000* (nuclear factor Y, subunit B13), *Glyma.10G0319000* (indole-3-acetic acid 7), *Glyma.10G056000* (SAUR-like auxin-responsive protein family), *Glyma.13G270800* (ubiquitin-conjugating enzyme 35), *Glyma.17G005900* (Pollen Ole e 1 allergen and extensin family protein), *Glyma.17G18500* (NAC domain containing protein 83), *Glyma.18G183600* (far-red elongated hypocotyl 1), and *Glyma.18G255300* (thioredoxin H-type 5). These results suggest that soybean epicotyl length may be regulated by multiple signaling pathways (**Table 1**).Additionally, none of these 10 cadidates were identified to be differentiated among wild soybean, landrace and improved cultivar (**Figure S1**).

**Table 1:** Gene based association of candidate genes.

Chr.	Physical position (bp)	Gene model	Trait	R^2^ (contribution rate)	Pvalue	Functional annotation
3	35863419	Glyma.03G142200	ELT_Q	0.5768	6.09167E-21	Ribosomal protein S10p/S20e family protein
4	15439303	Glyma.04G122400	ELT_Q	0.4336	8.01377E-07	DCD (Development and Cell Death) domain protein
4	26351924	Glyma.04G145000	ELS_Q	0.2279	4.20387E-22	nuclear factor Y, subunit B13
10	2738580	Glyma.10G031900	ELS_Q	0.5234	1.14306E-11	indole-3-acetic acid 7
10	5143580	Glyma.10G056000	ELT_Q	0.5294	5.84009E-32	SAUR-like auxin-responsive protein family
13	37284883	Glyma.13G270800	ELT_Q	1.5012	7.02497E-35	ubiquitin-conjugating enzyme 35
17	637613	Glyma.17G005900	ELT_Q	0.5942	5.10164E-10	Pollen Ole e 1 allergen and extensin family protein
17	23689587	Glyma.17G185000	ELS_Q	0.7863	4.96905E-13	NAC domain containing protein 83
18	44381201	Glyma.18G183600	ELS_QEI	2.1064	1.12718E-32	far-red elongated hypocotyl 1
18	44381201	Glyma.18G185300	ELS_QEI	2.1064	1.12718E-32	one helix protein

## Discussion

As an important feature of soybean variety, many studies indicated that EL affected 43.12% of seeds germination and 57.12% of seedlings emergence for soybean (Hanyu et al., 2020) estimated the genotypic determination coefficient of EL was more than 80% regardless of the evaluation period. (Matsuo et al., 2012) also obtained similar results. The genotypic determination coefficient was significantly related to inheritability, thus, it made the inference about genotypes possible (Vasconcelos et al., 2012; Hanyu et al., 2020). Through screening a large enough and reasonable gene database from more than 20,000 varieties, the SNPs and potential genes related to epicotyl traits were analyzed by GWAS technology. By elucidating the epicotyl related loci, it has a potential role in the study of early seed germination, seedling germination and stem strength of soybean.

To date, many seedling crop traits have been studied and elucidated, but epicotyl traits have been largely ignored and poorly studied. Four of Chr.2, Chr.4, Chr.7 and Chr.10 were identified in the F2 population of adzuki bean “Tokei1121” (T1121, long epimorph) and cultivar “Erimo167” (ordinary ectomorph) with EL associated SNP) (Mori et al., 2021). There are no reports on EL-related SNP sites in other plants. The genetic mechanism of the hypocotyl length trait (HL) has been extensively studied. SNP mapping of soybean root-related traits at seedling stage revealed that HL is regulated by multiple additive genes. Seven QTLs in HL associated with seedling photomorphology were identified by using recombinant inbred (RIL) populations obtained from biparental crosses between Patagonia (Pat) and Colombia (COL0) (Matsusaka et al., 2021). Compound spacer and epitaxial array localization methods were also used to identify HL loci associated with light-responsive quantitative traits (Wolyn et al., 2004). To pinpoint trait-associated loci, the combination of GWAS and transcriptome can be used to identify major genes affecting HL (Luo et al., 2017). These studies suggest that hypocotyl play a role in root growth and photomorphological responses. (Huang et al., 2006) studied the regulatory effect of brassinolide on epicotyl under low temperature conditions by proteomics. How xylan content in the gravitational bending direction of the epicotyl of adzuki bean affects its internal xylan content (Ikushima et al., 2008). Inhibitory effect of red light of the active form of phytochrome (Pfr) on epicotyl elongation in pea seedlings (Okoloko et al., 1970). These indicate that epicotyl play a non-negligible role in a variety of crops, especially dicotyledonous crops. Faced with this situation, this study used the soybean EL association panel to analyze the natural variation of epicotyl length and the related genetic structure, and analyzed the Hypothetically revealing a set of candidate genes controlling epicotyl development by GWAS analysis is undoubtedly a key step in filling in the relevant loci for epicotyl trait mapping.

### Putative genes involved in epicotyl length

Through the Arabidopsis annotation information, candidate gene phenotype contribution rate, and combining with Yu et al. (2022) Williams 82 and Jack transcriptome results of extremely different genes, we screened 22 potential genes from 103 hypothetical genes. These genes are located in SNP peak within 100Kb.10 significantly different candidate genes were identified by haplotype analysis, these genes were genotyped significantly and distinctly of ELS and ELT. *Glyma.03G142200* is a Ribosomal protein S10p/S20e family protein, proteins involved in photosynthesis (Bah et al., 2010). Wycoff found that a lectin protein, analogous to ribosomal proteins, is detected in roots, hypocotyls and leaves and involved in soybean nodule formation (Wycoff et al., 1997).

*Glyma.04G122400* DCD (Development and Cell Death) domain protein, thought to be involved in the hypersensitive response and programmed (Ludwig and Tenhaken, 2001, Enhaken et al., 2005), In previous studies, DCD domain proteins was believed to be involved in extracellular matrix or cytoskeleton proteins involved in growth and differentiation processes (Ichinose et al., 1990, Massimiliano et al., 2007).

*Glyma.04G145000* nuclear factor Y, subunit B13, Nuclear factor Y is one of the largest transcription factor gene families in plants, The NUCLEAR FACTOR Y (NF-Y) transcription factors are heterotrimeric complexes composed of NF-YA and histone-fold domain (HFD) containing NF-YB/NF-YC (Siriwardana et al., 2016), NF-Y subunits are emerging as transcriptional regulators with essential roles in diverse plant processes (Zanetti et al., 2010). playing key roles in development and in response to adverse environmental conditions (Nelson et al., 2007; Li et al., 2008)AtNF-YB6 (L1L) and AtNF-YB9 (LEC1) are involved in embryo development in seeds (Yamamoto et al., 2009). Overexpression of PdNF-YB7 in Arabidopsis exhibited earlier seedling establishment, longer primary roots, larger leaf areas, and increased photosynthetic rate that conferred drought tolerance and improved WUE in transgenic plants. In Arabidopsis, AtNF-YB3 plays an important role in the pro-motion of flowering specifically under inductive long-day photoperiodic conditions. Consistent with this, the overexpression of PdNF-YB7 in Arabidopsis caused earlier seedling germination time and enhanced the development of both vegetative and reproductive organs (Xiao et al., 2013), also found that overexpression of AtNF-YB2 enhanced primary root elongation due to a faster cell division and/or elongation(Ballif et al., 2011)

The soybean epicotyl is the basis for the formation of true leaves after seed germination, which ensures the normal development of seedlings, and the synthesis of related hormones is also important. The Glyma.10G056000 and Glyma.17G005900 encoding SAUR-like auxin-responsive protein and allergen and elongation protein, respectively, are annotated through multiple omics networks in the Arabidopsis genome (Depuydt and Vandepoele, 2021). Glyma.10G031900 encodes an indole-3-ACID 7 protein that functions as the principal component of the ABA-and auxin dependent reactions during post-germination seed growth (Belin et al., 2009). Glyma.13G270800 ubiquitin-conjugating enzyme 35, Previous studies have shown that ubiquitination plays important roles in plant abiotic stress responses, Protein ubiquitinations play crucial roles for numerous cellular processes such as cell growth, development, and response to diverse biotic and abiotic stresses. (Takahashi et al., 2009; Zhou et al., 2010), The ubiquitin-depen-dent protein degradation pathway is involved in photo-morphogenesis, hormone regulation, floral homeosis, senescence, and pathogen defense (Suzuki et al., 2002; Devoto et al., 2003).

Glyma.17G185000 NAC domain containing protein 83, The NAC (for NAM-ATAF1/2-CUC2) transcription factors constitute one of the largest transcription factor families in plant genomes (Ooka et al., 2004; Olsen et al., 2005b). Roles of many NAC transcription factors have been demonstrated in diverse develop- mental processes and plant responses to biotic and abiotic stresses, such apical meristem formation (Hibara et al., 2003), cell cycle control (Kim et al., 2006), AtNAC2 functioning in root development (He et al., 2005). cell divi-sion (Riechmann et al., 2000; Kim et al., 2006), NTM2 inte- grates auxin and salt signals in regulating Arabidopsis seed germination (Park et al., 2011), In Arabidopsis thaliana, 105 genes are predicted to encode NAC proteins (Ooka et al., 2004). Song et al. study found The highly homologous NAC transcription factors ANAC060, ANAC040 and ANAC089 regulate important transitions in the early phases of plant development. All three genes play a role in the interplay between the environment and the developmental switch that results in germination and/or seedling development (Song et al., 2022). For germination and seedling development to occur, the protein has to be released from the membrane, which for ANAC089 was shown to be directly affected by changes in the cellular redox status (Albertos et al., 2021).

Glyma.18g183600 far-red elongated hypocotyl 1, Phytochrome A (phyA) is the primary photoreceptor for mediating the far-red high irradiance response in Arabidopsis thaliana.FAR-RED ELONGATED HYPOCOTYL1 (FHY1) and its homolog FHY1-LIKE (FHL) define two positive regulators in the phyA signaling pathway (Shen et al., 2009). Most abundant in young seedlings in the dark.encodes FHY1 protein that mediates the transfer of phytochrome A (phyA) to the nucleus. Phytochrome A (phyA) acts as red and far red (FR) sensing photoreceptors to regulate plant growth and development (Helizon et al., 2018). Multiple metabolic pathways are required to regulate the length of soybean epicotyl (Clouse et al., 1992; Hao et al., 2014).

Glyma.18G185300 one helix protein, The cellular functions of two Arabidopsis (Arabidopsis thaliana) one-helix proteins, OHP1 and OHP2 (also named LIGHTHARVESTING-LIKE2 [LIL2] and LIL6, respectively, because they have sequence similarity to light-harvesting chlorophyll a/b-binding proteins), OHP1 and OHP2 play an essential role in chloroplast development as well as in vegetative growth, The photosynthetic capacity of ohp1-1 and ohp1-2 mutants also was decreased significantly (Myouga et al., 2018).The protein is localized to the thylakoid membrane and its transcript is transiently induced by exposure to high light conditions. increased expression of OHP1 is observed under light stress (Jansson et al., 2000). may constitute a novel mechanism of photoprotection in the plant photosynthetic apparatus (Psencik et al., 2020).

We speculate that traits during soybean domestication are gradually selected, and the priority traits are yield-related traits, such as seed size, oil content, and protein content (Wang et al., 2020). The epicotyl length involved in this study is not a major direct yield trait and therefore demonstrated weak signal of domestication selection.

In general, It is certain that most of the above candidate genes are related to the regulation of light and temperature, For example, the candidate gene Glyma.18G183600 is a phytochrome A (phyA) gene, which is the main photoreceptor mediating the far-red high-irradiation response in *Arabidopsis*. Cellular function of Glyma.18G185300 with sequence similarity to light-harvesting chlorophyll a/b binding protein, Glyma.03G142200 is a protein involved in photosynthesis, and the analysis results show that they are all involved in the growth and development of soybean epicotyl. This is consistent with the results that soybean epicotyl length is greatly affected by different environments. These results can be reflected from the haplotype analysis of ten candidate genes, which can be reflected in the significant differences in different environments (**Figure 9**).epicotyl However, further functional verification is needed to clarify the whole mechanism of action. More importantly, since the epicotyl is located in the country of cotyledons and true leaves, it is not only involved in seed germination and seedling growth, but also affects early morphogenesis of seedlings. Understanding and regulating the molecular regulatory network of epicotyl length has important guiding significance for crop breeding.

## Data availability statement

All whole genome sequencing data in this study have been deposited in the NCBI Sequence Read Archive under ac-cession number PRJNA681974.

## Author contributions

HH, and ML conceived the study and contributed to population development. YC, HW, JW, and BG contributed to phenotypic evaluation. HG, and HR analyzed the data. MY, and HG contributed to genotyping. LQ, and YH contributed to experimental design and writing the paper. All authors contributed to the article and approved the submitted version.

## Funding

This study was financially supported by the National Natural Science Foundation of China (32172005), Agricultural Science and Technology Innovation Program (ASTIP) of Chinese Academy of Agricultural Sciences.

## Conflict of interest

The authors declare that the research was conducted in the absence of any commercial or financial relationships that could be construed as a potential conflict of interest.

## Publisher’s note

All claims expressed in this article are solely those of the authors and do not necessarily represent those of their affiliated organizations, or those of the publisher, the editors and the reviewers. Any product that may be evaluated in this article, or claim that may be made by its manufacturer, is not guaranteed or endorsed by the publisher.

## References

[B1] Albertos P. Tatematsu K. Mateos I. Sánchez-Vicente I. Fernández-Arbaizar A. Nakabayashi K.. (2021). Redox feedback regulation of *ANAC089* signaling alters seed germination and stress response. Cell Rep. 35 (11), 109263. doi: 10.1016/j.celrep.2021.109263 34133931 PMC8220255

[B2] Albertos P. T atematsu K. Mateos I. Sánchez-Vicente I. Lorenzo O. (2021). Redox feedback regulation of *ANAC089* signaling alters seed germination and stress response. Cell Rep. 35 (11), 109263. doi: 10.1016/j.celrep.2021.109263 34133931 PMC8220255

[B3] Bah A. M. Sun H. Fei C. Zhou J. Dai H. Zhang G.. (2010). Comparative proteomic analysis of typha angustifolia leaf under chromium, cadmium and lead stress. J. Hazard Mater. 184 (1-3), 191–203. doi: 10.1016/j.jhazmat.2010.08.023 20817397

[B4] Ballif J. Endo S. Kotani M. Macadam J. Wu Y. (2011). Over-expression of *HAP3b* enhances primary root elongation in arabidopsis. Plant Physiol. Bioch. 49 (6), 579–583. doi: 10.1016/j.plaphy.2011.01.013 21316979

[B5] Bandelt H. Forster P. Röhl A. (1999). Median-joining networks for inferring intraspecific phylogenies. Mol. Biol. Evol. 16 (1), 37–48. doi: 10.1093/oxfordjournals.molbev.a026036 10331250

[B6] Belin C. Megies C. Hauserová E. Lopez-Molina L. (2009). Abscisic acid represses growth of the arabidopsis embryonic axis after germination by enhancing auxin signaling. Plant Cell. 21 (8), 2253–2268. doi: 10.1105/tpc.109.067702 19666738 PMC2751952

[B7] Bradbury P. J. Zhang Z. Kroon D. E. Casstevens T. M. Ramdoss Y. Buckler E. S. (2007). TASSEL: software for association mapping of complex traits in diverse samples. Bioinformatics 23 (19), 2633–2635. doi: 10.1093/bioinformatics/btm308 17586829

[B8] Camargos T. Campos N. Alves G. Ferreira S. Matsuo D. (2019). The effect of soil volume, plant density and sowing depth on soybean seedlings characters. Agron. Sci. Biotech. 5 (2), 47. doi: 10.33158/ASB.2019v5i2p47

[B9] Chaves M. V. A. Silva N. S. Silva R. H. O. Jorge G. L. Silveira I. C. Medeiros L. A.. (2017). Genotype x environment interaction and stability of soybean cultivars for vegetative-stage characters. Genet. Mol. Res. 16 (3). doi: 10.4238/gmr16039795 28973772

[B10] Chory J. Reinecke D. Sim S. Washburn T. Brenner M. (1994). A role for cytokinins in de-etiolation in arabidopsis. Plant Physiol. 104 (2), 339–347. doi: 10.1104/pp.104.2.339 12232085 PMC159204

[B11] Clement M. Snell Q. Walker P. Posada D. Crandall K. (2002). TCS: Estimating gene genealogies. parallel distributed Process. symposium Int. Proc. 2, 184.

[B12] Clouse S. D. Zurek D. M. McMorris T. C. Baker M. E. (1992). Effect of brassinolide on gene expression in elongating soybean epicotyls. Plant Physiol. 100 (3), 1377–1383. doi: 10.1104/pp.100.3.1377 16653132 PMC1075793

[B13] Contreras-Soto R. I. Mora F. Lazzari F. de Oliveira M. A. R. Scapim C. A. Schuster I. (2017). Genome-wide association mapping for flowering and maturity in tropical soybean: implications for breeding strategies. Breed Sci. 67 (5). doi: 10.3389/fpls.2017.01222 PMC5790042 29398937

[B14] Depuydt T. Vandepoele K. (2021). Multi-omics network-based functional annotation of unknown arabidopsis genes. Plant J. 108 (4), 1193–1212. doi: 10.1111/tpj.15507 34562334

[B15] Devoto A. Muskett P. R. Shirasu K. (2003). Role of ubiquitination in the regulation of plant defense against pathogens. Curr. Opin. Plant Biol. 6 (4), 307–311. doi: 10.1016/s1369-5266(03)00060-8 12873523

[B16] Dinka S. J. Campbell M. A. Demers T. Raizada M. N. (2007). Predicting the size of the progeny mapping population required to positionally clone a gene[J]. Genetics 176 (4), 2035–2054. doi: 10.1534/genetics.107.074377 17565938 PMC1950612

[B17] Enhaken R. Doerks T. Bork P. (2005). DCD – a novel plant specific domain in proteins involved in development and programmed cell death. BMC Bioinf. 6, 169. doi: 10.1186/1471-2105-6-169 PMC1182354 16008837

[B18] Flint-Garcia S. A. Thornsberry J. M. Th B. E. (2003). Structure of linkage disequilibrium in plants. Annu. Rev. Plant Biol. 54 (4), 357–374. doi: 10.1146/annurev.arplant.54.031902 14502995

[B19] Frances S. White M. J. Edgerton M. D. Jones A. M. Elliot R. C. Thompson W. F. (1992). Initial characterization of a pea mutant with light-independent photomorphogenesis. Plant Cell. 4 (12), 1519–1530. doi: 10.1105/tpc.4.12.1519 1467651 PMC160238

[B20] French N. Yu S. Biggs P. Holland B. Fearnhead P. Binney B.. (2014). “Evolution of campylobacter species in new Zealand,” in Campylobacter ecology and evolution. Eds. Sheppard S. K. Méric. G., 221–240. ISBN:978-1-908230-36-2.

[B21] Grove M. D. Spencer G. F. Rohwedder W. K. (1979). Brassinolide, a plant growth-promoting steroid isolated from brassica napus pollen[J]. Nature 281. doi: 10.1038/281216a0

[B22] Hanyu J. Costa S. Cecon P. Matsuo D. (2020). Genetic parameters estimate and characters analysis in phenotypic phase of soybean during two evaluation periods. Agron. Sci. Biotech. 6, 1–12. doi: 10.33158/ASB.r104.v6.2020

[B23] Hao H. P. He Z. Li H. Shi L. Tang Y. D. (2014). Effect of root length on epicotyl dormancy release in seeds of paeonia ludlowii. Tibetan peony. Ann. Bot. 113 (3), 443–452. doi: 10.1093/aob/mct273 24284815 PMC3906966

[B24] He J. X. Fujioka S. Li T. C. Kang S. G. Seto H. Takatsuto S.. (2003). Sterols regulate development and gene expression in *Arabidopsis* . Plant Physiol. 131 (3), 1258–1269. doi: 10.1104/pp.014605 12644676 PMC166886

[B25] Helizon H. Rösler-Dalton J. Gasch P. von Horsten S. Essen L. O. Zeidler M. (2018). Arabidopsis phytochrome a nuclear translocation is mediated by a far-red elongated hypocotyl 1-importin complex. Plant J. 96 (6), 1255–1268. doi: 10.1111/tpj.14107 30256472

[B26] He X. J. Mu R. L. Cao W. H. Zhang Z. G. Zhang J. S. Chen S. Y. (2005). *AtNAC2*, a transcription factor downstream of ethylene and auxin signaling pathways, is involved in salt stress response and lateral root development. Plant J. 44 (6), 903–916. 16359384 10.1111/j.1365-313X.2005.02575.x

[B27] Hibara K. Takada S. Tasaka M. (2003). *CUC1* gene activates the expression of SAM-related genes to induce adventitious shoot formation. Plant J. 36 (5), 687–696. 14617069 10.1046/j.1365-313x.2003.01911.x

[B28] Huang B. Chu C. H. Chen S. L. Juan H. F. Chen Y. M. (2006). A proteomics study of the mung bean epicotyl regulated by brassinosteroids under conditions of chilling stress. Cell Mol. Biol. Lett. 11 (2), 264–278. doi: 10.2478/s11658-006-0021-7 16847571 PMC6275966

[B29] Ichinose A. Bottenus R. E. Davie E. W. (1990). Structure of transglutaminases. J. Biol. Chem. 265 (23), 13411–13414. doi: 10.1016/0008-6215(90)80036-3 1974250

[B30] Ikushima T. Soga K. Hoson T. Shimmen T. (2008). Role of xyloglucan in gravitropic bending of azuki bean epicotyl. Physiol. Plant 132 (4), 552–565. doi: 10.1111/j.1399-3054.2007.01047 18248506

[B31] Jansson S. Andersson J. Kim S. J. Jackowski G. (2000). An *Arabidopsis* thaliana protein homologous to cyanobacterial high-light-inducible proteins. Plant Mol. Biol. 42 (2), 345–351. doi: 10.1023/a:1006365213954 10794534

[B32] Jia Q. Xiao Z. X. Wong F. L. Sun S. Liang K. J. Lam H. M. (2017). Genome-wide analyses of the soybean f-box gene family in response to salt stress. Int. J. Mol. Sci. 18 (4), 818. doi: 10.3390/ijms18040818 28417911 PMC5412402

[B33] Kang H. M. Sul J. H. Service S. K. Zaitlen N. A. Kong S. Y. Freimer N. B.. (2010). Variance component model to account for sample structure in genome-wide association studies. Nat. Genet. 42 (4), 348–354. doi: 10.1038/ng.548 20208533 PMC3092069

[B34] Kang H. M. Zaitlen N. A. Wade C. M. Kirby A. Heckerman D. Daly M. J.. (2008). Efficient control of population structure in model organism association mapping. Genetics 178 (3), 1709–1723. doi: 10.1534/genetics.107.080101 18385116 PMC2278096

[B35] Kan G. Zhang W. Yang W. Ma D. Zhang D. Hao D.. (2015). Association mapping of soybean seed germination under salt stress. Mol. Genet. Genomics 290 (6), 2147–2162. doi: 10.1007/s00438-015-1066-y 26001372

[B36] Kim Y. S. Kim S. G. Park J. E. Park H. Y. Lim M. H. Chua N. H.. (2006). A membrane-bound NAC transcription factor regulates cell division in *Arabidopsis* . Plant Cell. 18 (11), 3132–3144. doi: 10.1105/tpc.106.043018 17098812 PMC1693948

[B37] Liang H. Yu Y. Yang H. Zhang H. Wei D. Cui W.. (2014). Epistatic effects and quantitative trait Loci(QTL) x Environment(QE) interaction effects for yield per plot and botanical traits in soybean. Chin. Bull. Bot. doi: 10.3724/SP.J.1259.2014.00273

[B38] Li Y. H. Li D. L. Jiao Y. Q. Schnable J. C. Li Y. F. Li H. H.. (2020a). Identification of loci controlling adaptation in Chinese soya bean landraces *via* a combination of conventional and bioclimatic GWAS. Plant Biotechnol. J. 18 (2), 389–401. doi: 10.1111/pbi.13206 31278885 PMC6953199

[B39] Lipka A. E. Tian F. Wang Q. Peiffer J. Li M. Bradbury P. J.. (2012). GAPIT:genome association and prediction integrated tool. Bioinf. 15; 28 (18), 2397–2399. doi: 10.1093/bioinformatics/bts444 22796960

[B40] Lippert C. Listgarten J. Liu Y. Kadie C. M. Davidson R. I. Heckerman D. (2011). FaST linear mixed models for genome-wide association studies. Nat. Methods 8 (10), 833–835. doi: 10.1038/nmeth.1681 21892150

[B41] Li Y. Qin C. Wang L. Jiao C. Z. Hong H. L. Tia Y.. (2022d). Genome-wide signatures of geographic expansion and breeding process in soybean. Sci. China Life Sci. 19. doi: 10.1007/s11427-022-2158-7 35997916

[B42] Li Y. H. Reif J. C. Hong H. L. Li H. H. Liu Z. X. Ma Y. S.. (2018). Genome-wide association mapping of QTL underlying seed oil and protein contents of a diverse panel of soybean accessions. Plant Sci. 266, 95–101. doi: 10.1016/j.plantsci.2017.04.013 29241572

[B43] Liu M. Tan X. Yang Y. Liu P. Zhang X. Zhang Y.. (2020). Analysis of the genetic architecture of maize kernel size traits by combined linkage and association mapping. Plant Biotechnol. J. 18 (1), 207–221. doi: 10.1111/pbi.13188 31199064 PMC6920160

[B44] Li M. Zhang Y. W. Xiang Y. Liu M. H. Zhang Y. M. (2022c). IIIVmrMLM: The r and c++ tools associated with 3VmrMLM, a comprehensive GWAS method for dissecting quantitative traits. Mol. Plant 15 (8), 1251–1253. doi: 10.1016/j.molp.2022.06.002 35684963

[B45] Li M. Zhang Y. W. Zhang Z. C. Xiang Y. Liu M. H. Zhou Y. H.. (2022b). ). a compressed variance component mixed model for detecting QTNs and QTN-by-environment and QTN-by-QTN interactions in genome-wide association studies. Mol. Plant 15 (4), 630–650. 35202864 10.1016/j.molp.2022.02.012

[B46] Li X. Zheng H. Wu W. Liu H. Wang. J. Jia Y.. (2020). QTL mapping and candidate gene analysis for alkali tolerance in japonica rice at the bud stage based on linkage mapping and genome-wide association study. Rice (N Y). 1316 (1), 48. doi: 10.1186/s12284-020-00412-5 32676742 PMC7364718

[B47] Ludwig A. A. Tenhaken R. (2001). A new cell wall located n-rich protein is strongly induced during the hypersensitive response in *Glycine max* l. Eur. J. Plant Pathol. 107, 323–336. doi: 10.1023/A:1011202225323

[B48] Luo X. Xue Z. Ma C. Hu K. Zeng Z. Dou S.. (2017). Joint genome-wide association and transcriptome sequencing reveals a complex polygenic network underlying hypocotyl elongation in rapeseed (*Brassica napus* l.). Sci. Rep. 7, 41561. doi: 10.1038/srep41561 28139730 PMC5282501

[B49] Mathur J. Molnár G. Fujioka S. Takatsuto S. Sakurai A. Yokota T.. (1998). Transcription of the arabidopsis CPD gene, encoding a steroidogenic cytochrome P450, is negatively controlled by brassinosteroids. Plant J. 14 (5), 593–602. doi: 10.1046/j.1365-313x 9675902

[B50] Matsuo E. Sediyama T. Cruz C. D. Oliveira R. (2012). Estimates of the genetic parameters, optimum sample size and conversion of quantitative data in multiple categories for soybean genotypes. Acta Sci. Agron. 34 (3), 265–273. doi: 10.4025/actasciagron.v34i3.14015

[B51] Matsusaka D. Filiault D. Sanchez D. H. Botto J. F. (2021). Ultra-High-Density QTL marker mapping for seedling photomorphogenesis mediating arabidopsis establishment in southern patagonia. Front. Plant Sci. 12. doi: 10.3389/fpls.2021.677728 PMC8343176 34367202

[B52] Mori M. Maki K. Kawahata T. Kawahara D. Kato Y. Yoshida T.. (2021). Mapping of QTLs controlling epicotyl length in adzuki bean (*Vigna angulari*s). Breed Sci. 71 (2), 208–216. doi: 10.1270/jsbbs.20093 34377069 PMC8329883

[B53] Myouga F. Takahashi K. Tanaka R. Nagata N. Shinozaki K. (2018). Stable accumulation of photosystem II requires *ONE-HELIX PROTEIN1 (OHP1)* of the light harvesting-like family[J]. Plant Physiol. 176 (3), 01782.2017. doi: 10.1104/pp.17.01782 PMC5841713 29438089

[B54] Okoloko G. E. Lewis L. N. Reid B. R. (1970). Changes in nucleic acids in phytochrome-dependent elongation of the alaska pea epicotyl. Plant Physio. 46 (5), 660–665. doi: 10.1104/pp.46.5.660 PMC396657 16657526

[B55] Olsen A. N. Ernst H. A. Leggio L. L. Skriver K. (2005). DNA-Binding specificity and molecular functions of NAC transcription factors. Plant Sci. 169 (4), 785–797. doi: 10.1016/j.plantsci.2005.05.035

[B56] Ooka H. Satoh K. Doi K. T Nagata T. S. Kikuchi S. (2004). Comprehensive analysis of NAC family genes in *Oryza sativa* and *Arabidopsis thaliana* . DNA Res. 10 (6), 239–247. doi: 10.1093/dnares/10.6.239 15029955

[B57] Park J. Kim Y. S. Kim S. G. Jung J. H. Woo J. C. Park C. M. (2011). Integration of auxin and salt signals by the *NAC* transcription factor *NTM2* during seed germination in *Arabidopsis* . Plant Physiol. 156 (2), 537–549. doi: 10.1104/pp.111.177071 21450938 PMC3177257

[B58] Psencik J. Hey D. Grimm B. Lokstein H. (2020). Photoprotection of photosynthetic pigments in plant one-helix protein 1/2 heterodimers. J. Phys. Chem. Lett. 11 (21), 9387–9392. doi: 10.1021/acs.jpclett.0c02660 33095593

[B59] Riechmann J. L. Heard J. Martin G. Reuber L. Jiang C. Z. Keddie J.. (2000). *Arabidopsis* transcription factors: Genome-wide comparative analysis among eukaryotes. Science 290 (5499), 2105–2110. doi: 10.1126/science.290.5499.2105 11118137

[B60] Segura V. Vilhjálmsson B. J. Platt A. Korte A. Seren Ü. Long Q.. (2012). An efficient multi-locus mixed-model approach for genome-wide association studies in structured populations. Nat. Genet. 44 (7), 825–830. doi: 10.1038/ng.2314 22706313 PMC3386481

[B61] Seyedi M. Selstam E. Timko M. P. Sundqvist C. (2001). The cytokinin 2-isopentenyladenine causes partial reversion to skotomorphogenesis and induces formation of prolamellar bodies and protochlorophyllide657 in the lip1 mutant of pea. Physiol. Plant 112 (2), 261–272. doi: 10.1034/j.1399-3054 11454232

[B62] Shen Y. Zhou Z. Feng S. Li J. Tan-Wilson A. Qu L. J.. (2009). Phytochrome a mediates rapid red light-induced phosphorylation of arabidopsis FAR-RED *ELONGATED HYPOCOTYL1* in a low fluence response. Plant Cell. 21 (2), 494–506. doi: 10.1105/tpc.108.061259 19208901 PMC2660616

[B63] Song Q. Hyten D. L. Jia G. Quigley C. V. Fickus E. W. Nelson R. L.. (2013). Development and evaluation of SoySNP50K, a high-density genotyping array for soybean. PloS One 8 (1), e54985. doi: 10.1371/journal.pone.0054985 23372807 PMC3555945

[B64] Song S. Willems L. Jiao A. Zhao T. Eric S. M. Leónie B. (2022). The membrane associated NAC transcription factors *ANAC060* and *ANAC040* are functionally redundant in the inhibition of seed dormancy in arabidopsis thaliana. J. Exp. Bot. 73 (16), 5514–5528. doi: 10.1093/jxb/erac232 35604925 PMC9467645

[B65] Sui M. Jing Y. Li H. Zhan Y. Luo J. Teng W.. (2020). Identification of loci and candidate genes analyses for tocopherol concentration of soybean seed. Front. Plant Sci. 11. doi: 10.3389/fpls.2020.539460 PMC7509058 33013963

[B66] Suzuki G. Yanagawa Y. Kwok S. F. Matsui M. Deng X. W. (2002). *Arabidopsis COP10* is a ubiquitin-conjugating enzyme variant that acts together with *COP1* and the *COP9* signalosome in repressing photomorphogenesis. Genes Dev. 16 (5), 554–559. doi: 10.1101/gad.964602 11877375 PMC155353

[B67] Takahashi H. Nozawa A. Seki M. Shinozaki K. Endo Y. Sawasaki T. (2009). A simple and high-sensitivity method for analysis of ubiquitination and polyubiquitination based on wheat cell-free protein synthesis. BMC Plant Biol. 9 (1), 39. doi: 10.1186/1471-2229-9-39 19348673 PMC2674041

[B68] Theodorsson N. E. (1986). Kruskal-Wallis test: BASIC computer program to perform nonparametric one-way analysis of variance and multiple comparisons on ranks of several independent samples. Comput. Meth Prog. Bio. 23 (1), 57–62. doi: 10.1016/0169-2607(86)90081-7 3638187

[B69] Wang S. B. Feng J. Y. Ren W. L. Huang B. Zhou L. Wen Y. J.. (2016). Improving power and accuracy of genome-wide association studies *via* a multi-locus mixed linear model methodology. Sci. Rep. 6, 19444. doi: 10.1038/srep19444 26787347 PMC4726296

[B70] Wang X. Guan P. Xin M. Wang Y. Chen X. Zhao A.. (2021). Genome-wide association study identifies QTL for thousand grain weight in winter wheat under normal- and late-sown stressed environments. Theor. Appl. Genet. 134 (1), 143–157. doi: 10.1007/s00122-020-03687-w 33030571

[B71] Wang S. Liu S. Wang J. Yokosho K. Zhou B. Yu Y. C.. (2020). Simultaneous changes in seed size, oil content and protein content driven by selection of *SWEET* homologues during soybean domestication. Natl. Sci. Rev. 7 (11), 1776–1786. doi: 10.1093/nsr/nwaa110 34691511 PMC8290959

[B72] Wen Y. J. Zhang H. Ni Y. L. Huang B. Zhang J. Feng J. Y.. (2017). Methodological implementation of mixed linear models in multi-locus genome-wide association studies. Brief Bioinform. 19 (4), 700–712. doi: 10.1093/bib/bbw145 PMC6054291 28158525

[B73] Wolyn D. J. Borevitz J. O. Loudet O. Schwartz C. Maloof J. Ecker J. R.. (2004). Light-response quantitative trait loci identified with composite interval and eXtreme array mapping in *Arabidopsis thaliana* . Genetics 167 (2), 907–917. doi: 10.1534/genetics.103.024810 15238539 PMC1470895

[B74] Wu D. P. Li D. M. Zhao X. Zhan Y. H. Teng W. L. Qiu L. J.. (2020). Identification of a candidate gene associated with isoflavone content in soybean seeds using genome-wide association and linkage mapping. Plant J. 104 (4), 950–963. doi: 10.1111/tpj.14972 32862479

[B75] Wycoff K. L. Rhijn P. V. Hirsch A. M. (1997). The ribosomal protein P0 of soybean (*Glycine max* l. merr.) has antigenic cross-reactivity to soybean seed lectin. Plant Mol. Biol. 34 (2), 295–306. doi: 10.1023/a:1005817114562 9207845

[B76] Xiao H. Tang S. An Y. Zheng D. C. Xia X. L. Yin W. L. (2013). Overexpression of the poplar NF-YB7 transcription factor confers drought tolerance and improves water-use efficiency in arabidopsis. J. Exp. Bot. 64 (14), 4589–4601. doi: 10.1093/jxb/ert262 24006421 PMC3808328

[B77] Yamamoto A. Kagaya Y. T oyoshima R. Kagaya M. T akeda S. Hattori T. (2009). *Arabidopsis* NF-YB subunits LEC1 and LEC1-LIKE activate transcription by interacting with seed-specific ABRE-binding factors. Plant J. 58 (5), 843–856. doi: 10.1111/j.1365-313X.2009.03817.x 19207209

[B78] Yan L. Hofmann N. Li S. Ferreira M. E. Song B. Jiang G.. (2017). Identification of QTL with large effect on seed weight in a selective population of soybean with genome-wide association and fixation index analyses. BMC Genomics 18 (1), 529. doi: 10.1186/s12864-017-3922-0 28701220 PMC5508781

[B79] Yu J. Pressoir G. Briggs W. H. Vroh B. I. Yamasaki M. Doebley J. F.. (2006). A unified mixed-model method for association mapping that accounts for multiple levels of relatedness. Nat. Genet. 38 (2), 203–208. doi: 10.1038/ng1702 16380716

[B80] Yu Y. Zhang H. Long Y. P. Shu Y. Zhai J. X. (2022). PPRD: a comprehensive online database for expression analysis of ~45,000 plant public RNA-seq libraries. Plant Biotechnol. J. 20 (5), 806–808. doi: 10.1111/pbi.13798 35218297 PMC9055819

[B81] Zhang Z. Ersoz E. Lai C. Q. Todhunter R. J. Tiwari H. K. Gore M. A.. (2010). Mixed linear model approach adapted for genome-wide association studies. Nat. Genet. 42 (4), 355–360. doi: 10.1038/ng.546 20208535 PMC2931336

[B82] Zhang Y. M. Mao Y. Xie C. Smith H. Luo L. Xu S. (2005). Mapping quantitative trait loci using naturally occurring genetic variance among commercial inbred lines of maize (*Zea mays* l.). Genetics 169 (4), 2267–2275. doi: 10.1534/genetics.104.033217 15716509 PMC1449576

[B83] Zhang J. P. Singh A. P. Mueller D. S. Singh A. K. (2015). Genome-wide association and epistasis studies unravel the genetic architecture of sudden death syndrome resistance in soybean. Plant J. 84 (6), 1124–1136. doi: 10.1111/tpj.13069 26561232

[B84] Zhang T. Wu T. Wang L. Jiang B. Zhen C. Yuan S.. (2019). A combined linkage and GWAS analysis identifies QTLs linked to soybean seed protein and oil content. Int. J. Mol. Sci. 20 (23), 5915. doi: 10.3390/ijms20235915 31775326 PMC6928826

[B85] Zhao X. Han Y. Li Y. Liu D. Sun M. Zhao Y.. (2015). Loci and candidate gene identification for resistance to sclerotinia sclerotiorum in soybean (*Glycine max l.* merr.) *via* association and linkage maps. Plant J. 82 (2), 245–255. doi: 10.1111/tpj.12810 25736370

[B86] Zhao S. P. Lu D. Yu T. F. Ji Y. J. Zheng W. J. Zhang S. X.. (2017). Genome-wide analysis of the YABBY family in soybean and functional identification of *GmYABBY10* involvement in high salt and drought stresses. Plant Physiol. Biochem. 119, 132–146. doi: 10.1016/j.plaphy.2017.08.026 28866235

[B87] Zhao X. Teng W. L. Li Y. H. Liu D. Y. Cao G. L. Li D. M.. (2017). Loci and candidate genes conferring resistance to soybean cyst nematode HG type 2.5.7. BMC Genomics 14, 18(1):462. doi: 10.1186/s12864-017-3843-y PMC5471737 28615053

[B88] Zhou G. A. Chang R. Z. Qiu L. J. (2010). Overexpression of soybean ubiquitin-conjugating enzyme gene GmUBC2 confers enhanced drought and salt tolerance through modulating abiotic stress-responsive gene expression in arabidopsis. Plant Mol. Biol. 72 (s4-5), 357–367. doi: 10.1007/s11103-009-9575-x 19941154 PMC2816239

[B89] Zhou X. Stephens M. (2012). Genome-wide efficient mixed-model analysis for association studies. Nat. Genet. 44 (7), 821–824. doi: 10.1038/ng.2310 22706312 PMC3386377

