# Supplementary figures and images for: Genome-wide association studies for soybean epicotyl length in two environments using 3VmrMLM
Source: Front Plant Sci. 2022 Nov 14;13:1033120. doi: 10.3389/fpls.2022.1033120 (PMC9704727; doi:10.3389/fpls.2022.1033120)

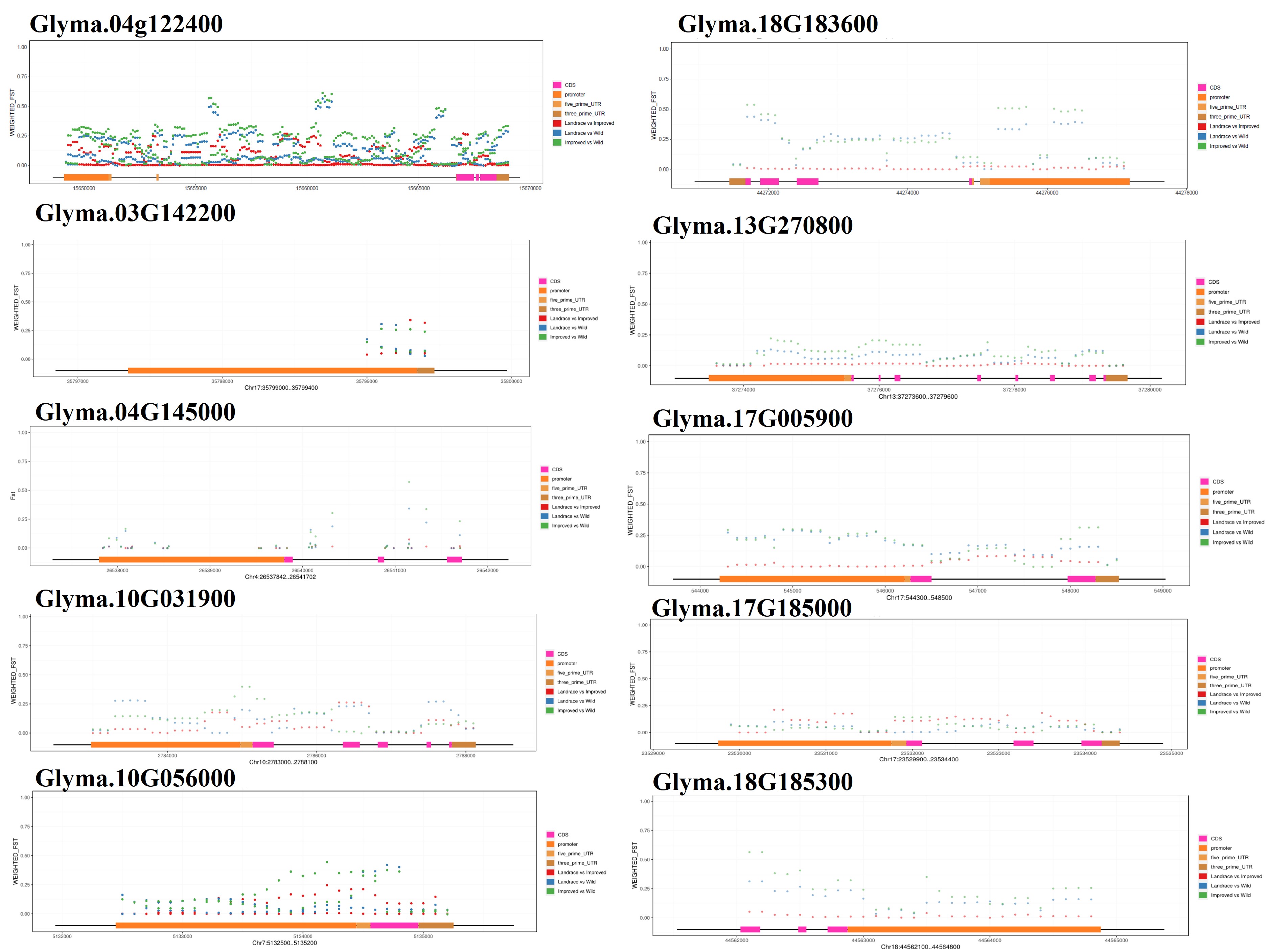

Supplement: Supplementary file 2 [file Image_1.tiff]
